# Association between quality of life and resilience in infertile patients: a systematic review

**DOI:** 10.3389/fpubh.2024.1345899

**Published:** 2024-02-27

**Authors:** Kexian Liu, Shanshan Dou, Wei Qin, Di Zhao, Wei Zheng, Dan Wang, Caixia Zhang, Yichun Guan, Peiling Tian

**Affiliations:** ^1^Third Affiliated Hospital of Zhengzhou University, Zhengzhou, Henan Province, China; ^2^Zhejiang University, Hangzhou, Zhejiang Province, China

**Keywords:** quality of life, resilience, infertility, association, systematic review

## Abstract

This systematic review was carried out to describe QoL and resilience in infertile patients, as well as the relationship between them, and to give a theoretical foundation for clinical practice. The databases of CNKI, Wanfang data, VIP database, PubMed, Web of Science, and Embase were searched without a time limit. A narrative synthesis of relevant articles was undertaken. This systematic review was registered on PROSPERO in advance. Of 21 studies eligible for inclusion in this review, 13 focused on the relationship between QoL and resilience, 5 on QoL influencing factors (resilience included), and 3 on mediation effect analysis on mental health (resilience as a mediator). Resilience can significantly predict the QoL of infertile patients. It seems plausible that more resilient couples will be less vulnerable to the stress of infertility. A global consortium of infertile population research could make cross-cultural comparisons of QoL and resilience possible. Future research should focus on resilience therapies.

**Systematic review registration:**

This systematic review was registered on PROSPERO in advance (CRD42023414706).

## 1 Introduction

When a clinical pregnancy cannot be established after 12 months of regular, unprotected sexual activity or when a person’s ability to reproduce, either alone or with a partner, is impaired, the condition is referred to as infertility (1). One in six persons worldwide suffer from infertility, which affects 17.8 and 16.5% of people in high- and low- or middle-income nations, respectively (2). It is now a significant global public health issue.

Negative emotional experiences, including as anxiety, depression, and stress, are extremely common among infertile people (3). This is linked to a number of complex elements, including traditional social conceptions and the economic cost of treatment (4). Negative emotions can also exacerbate infertility and negatively impact treatment success (5). Previous research has found that infertility is frequently connected with poor quality of life (QoL) (6). The physiology, psychology, social interactions, and the medical environment all contribute to the quality of life (QoL) of infertile people. There were numerous techniques used to assess QoL, and the Fertility quality of life (FertilQoL) was designed and widely used in infertile populations with poor QoL (7, 8).

Individuals’ resilience is described as their mental ability to withstand and adapt to life-threatening experiences (9). When stressors are removed, a person with resilience adapts to changes flexibly and returns to recovery easily. In contrast, people with weaker resilience are less able to adjust to new circumstances. Couples who have a higher QoL despite infertility are more mentally tolerant of excessive stress (10). We can hypothesize that resilience acts as a protective factor in increasing QoL. Previous research has focused on the relationship between QoL and resilience. However, the findings are inconclusive, and there are disparities in how much emphasis the studies place on men and women. Several studies have reported that levels of QoL are higher in more resilient populations (6, 11), while others have found no significant correlation between QoL and resilience (12). This contradiction in the available literature may be attributable, at least in part, to variations in the scales and subjects utilized in these studies.

Consequently, it is imperative to conduct a comprehensive review of the existing literature on QoL and resilience in infertile patients. This systematic review was carried out to describe QoL and resilience in infertile patients, as well as the relationship between them, and to give a theoretical foundation for clinical practice. The literature incorporated in this article encompasses diverse research designs and data analytical techniques, which are advantageous in elucidating the intricate interplay among biological, cognitive, and social determinants. This understanding could potentially facilitate the development of interventions aimed at assisting infertile patients in leading well-being lives.

## 2 Review

### 2.1 Objective

This retrospective article attempts to provide supporting evidence by summarizing the relationship between quality of life and resilience in infertile patients. Potential countermeasures were investigated in order to design relevant future interventions for patients.

### 2.2 Methods

#### 2.2.1 Design

This systematic review was registered on PROSPERO in advance. The System Review and Meta-Analysis Preferred Reporting Project (PRISMA) guidelines were used to select publications for inclusion (13). To assess the quality of the papers, the Agency for Healthcare Research and Quality (AHRQ) checklist (14) was employed.

#### 2.2.2 Search strategy

We did a thorough literature search using the CNKI, Wanfang data, VIP database, PubMed, Web of Science, and Embase databases from their inception through April 2023. Using free words and a mix of logical operators, synonyms of the search phrases were obtained after multiple attempts and changes. Supplementary Table S1 displays the specific search formulas for each database. In addition, we analyzed the references of the included studies and manually obtained them as needed to locate other potential studies that satisfied the inclusion criteria.

#### 2.2.3 Inclusion criteria

Our systematic review included research that met the following criteria: (1) observational studies, (2) patients with infertility included, (3) the link between quality of life and resilience documented, and (4) papers published in Chinese or English. Retrieved dissertations that met the inclusion criteria were also considered. Interventional studies, reviews, qualitative research, case reports and letters to the editor, preprint studies, and lack of access to the full text of articles were all excluded. Studies targeted at patients with known mental disorders were excluded.

#### 2.2.4 Study selection

**Figure 1:**
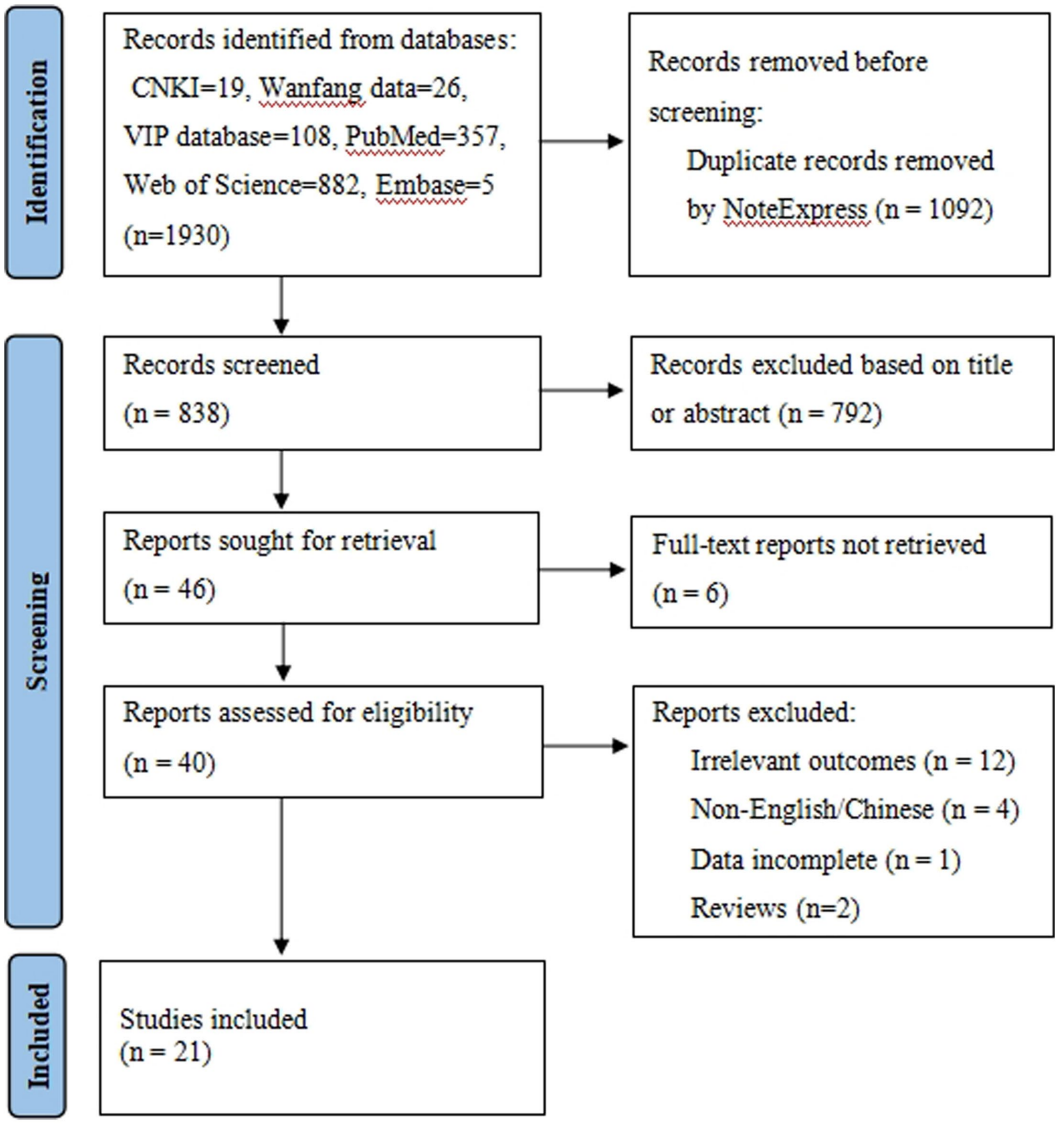
PRISMA flow diagram.

NoteExpress was used to remove duplicate entries from the database search. The first author conducted the searches and completed the initial screening from the titles and abstracts. The full texts of all potential titles were independently reviewed by 2 authors, and an article selection decision was made based on the inclusion criteria. The reasons for the article’s exclusion were documented. A third reviewer resolved any disagreements between the two reviewers about the inclusion of an article. The PRISMA flow diagram of our study selection procedure is shown in Figure 1 (13).

#### 2.2.5 Data extraction

Two reviewers worked independently to retrieve data. Disagreements were settled by enlisting the help of a third author to review the data. All of the included studies were cross-sectional. The data extraction form included the following items: (1) the first author, (2) the year of publication, (3) the study location, (4) the study period, (5) the settings, (6) the study populations, (7) the sampling strategies, (8) the sample sizes, (9) patient demographics (mean/median age), (10) screening instruments and scores, (11) data analysis, and (12) the research findings.

#### 2.2.6 Outcomes and measures

The primary findings of this systematic review were pooled ratings of quality of life and resilience, as well as the relationship between them in infertility patients utilizing self-questionnaires. Fertility quality of life (FertiQoL) (13), The World Health Organization Quality of Life-BREF (WHOQOL-BREF) (1998) (15), Quality of Life questionnaire for Infertile Couples (QoLICQ) (16), Connor-Davidson Resilience Scale (CD-RISC) (9), 10-item Connor-Davidson Resilience Scale (CD-RISC-10) (17), The 14-item Resilience Scale (RS-14) (11), and The Resilience Scale (RS) (18) were all included.

#### 2.2.7 Quality assessment

Two reviewers independently assessed the quality of the papers using the AHRQ guideline (14). The AHRQ checklist provides a framework for evaluating reporting quality and bias risk in 5 domains: selection, implementation, measurement, reporting, and follow-up. This guide contains 11 items totaling 11 points. If an item satisfied the criteria, it received 1 point. If the description was negative or ambiguous, it received a score of zero. A score of ≥8 indicates that the article is of good quality. A medium-quality article has a score between 4 and 7. A score of ≤3 indicates a low-quality article.

## 3 Results

### 3.1 Characteristics of the included literature

There were 21 cross-sectional studies included in this study (Figure 1). All of them were published in peer-reviewed publications. The papers featured 7,892 people from 5 different nations, with 2,678 males and 5,214 females. There were 10 studies that concentrated on women (12, 19–27), 9 studies that recruited both men and women (6, 8, 10, 11, 28–32), and just 2 studies that focused on men (4, 33). Twenty studies were carried out in Asia [China (4, 12, 19–27, 30–33), Pakistan (11, 28), Iran (6, 10), and South Korea (8)], with one carried out in Europe [Germany (29)]. This paper included 21 studies, the majority of which focused on present QoL and resilience, the relationship between QoL and resilience, and other psychological issues in infertile patients. In 21 cross-sectional studies, 13 studies focused on the correlation between QoL and resilience (4, 6, 8, 19, 22–29, 32), 5 on QoL influencing factors (10, 21, 30, 31, 33), and 3 on mediation effect analysis on mental health (11, 12, 20). Tables 1, 2 show the characteristics and outcomes.

**Table 1:** Characteristics of included studies.

First author	Year published	Study location	Study period	Setting	Study population	Sampling strategy	Sample size (M/F)	Age Mean ± SD /Median (Range)	Assessment scales and scores				Data analysis	*r*/*β*
									QoL	Scores Mean ± SD /Median (Range)	Resilience	Scores Mean ± SD /Median (Range)		
Jiang (18)	2022	China	Dec. 2019–Dec. 2020	Hospital	Patients with RIF	–	95 (0/95)	–	FertiQoL	68.63 ± 2.63	CD-RISC	64.97 ± 11.25	CA&MLR	−/0.459
Lian (10)	2022	China	May.2020–Apr. 2021	Hospital	Patients with RIF	–	193 (0/193)	–	FertiQoL	59.0 (25.0–88.1)	CD-RISC	28.0 (0.0–100.0)	CA&MLR	0.60/0.33
Xie (31)	2022	China	May.2021–Oct. 2021	Hospital	Patients with asthenospermia	Convenience	198 (198/0)	–	FertiQoL	64.73 ± 11.04	CD-RISC	66.41 ± 12.99	CA&MLR	0.479/0.426
Zhou (33)	2022	China	Jun. 2018–Mar. 2019	Hospital	Infertile men	–	335 (335/0)	31 ± 12	FertiQoL	53.03 ± 17.05	CD-RISC	63.8 ± 14.8	CA&MLR	0.45/0.62
Mo (5)	2021	China	Sept.2019–Dec. 2020	Hospital	Infertile women	–	400 (0/400)	30.44 ± 3.53	FertiQoL	68.31 ± 12.32	CD-RISC-10	26.37 ± 6.64	LCA	–
Zhou (1)	2023	China	Oct. 2019–May. 2021	Hospital	Infertile patients	Random	566 (271/295)	–	FertiQoL	59.74 ± 12.82	CD-RISC	59.28 ± 10.13	CA&MLR	0.473/0.582
Dong (6)	2021	China	Dec. 2019–Dec. 2020	Hospital	Patients with PCOS	Convenience	134 (0/134)	25.46 ± 3.91	WHOQOL-BREF	2.56 ± 0.47	CD-RISC	62.40 ± 14.48	CA&MM	0.592/−
Liu (13)	2020	China	Nov.2019–Feb. 2020	Hospital	Patients with RIF	Convenience	125 (0/125)	–	FertiQoL	58.18 ± 17.87	CD-RISC	65.09 ± 15.14	CA&MLR	0.605/0.081
Zhou (14)	2020	China	Oct. 2018–Apr. 2019	Hospital	Infertile patients	Convenience	100 (25/75)	M:33.52 ± 5.30 F:33.57 ± 6.03	FertiQoL	65.72 ± 11.87	CD-RISC	59.82 ± 20.78	CA	0.434/−
Liu (30)	2019	China	2017–2018	Hospital	Infertile women	Convenience	548 (0/548)	32.8 ± 4.42	FertiQoL	79.55 ± 16.78	CD-RISC	56.99 ± 14.87	CA&MLR&MM	0.285/0.208
Zhang (4)	2019	China	Apr. 2017–Jun. 2017	Hospital	Infertile women	–	116 (0/116)	33.22 ± 5.42	FertiQoL	67.05 ± 14.88	CD-RISC	68.84 ± 13.25	CA	0.463/−
Zhou (34)	2019	China	Apr. 2016–Apr. 2017	Hospital	Infertile women	–	576 (0/576)	31.32 ± 5.71	FertiQoL	58.54 ± 13.04	CD-RISC	60.23 ± 15.20	CA&MLR	0.227/0.673
Zhang (35)	2017	China	Jun. 2016–Nov. 2016	Hospital	Infertile women	Convenience	310 (0/310)	32.47 ± 4.99	FertiQoL	67.47 ± 11.93	CD-RISC	66.52 ± 14.32	CA&MM	0.434/−
Wang (25)	2022	China	Oct. 2020–Jan. 2021	Hospital	Infertile couples	Convenience	856 (428/428)	M:32.42 ± 5.19 F:31.00 ± 1.24	FertiQoL	M:67.36 ± 12.44 F:63.21 ± 12.18	CD-RISC-10	M:29.78 ± 7.31 F:26.89 ± 7.11	CA&APIM	0.499/0.284^a^ 0.373/−^b^ 0.192/0.104^c^ 0.143/−^d^
Bhamani (23)	2022	Pakistan	2017–2018 9 months	Hospital	Infertile couples	Purposive	668 (334/334)	M:35.53 ± 6.67 F:30.87 ± 6.12	FertiQoL	M:81.66 ± 12.09 F:70.44 ± 15.69	RS-14	M:77.64 ± 8.56 F:76.19 ± 8.69	CA	M&F: 0.161&0.149/−
Vatanparast (12)	2022	Iran	Mar. 2017–Jun. 2017	Hospital	Infertile couples	Convenience Simple random	404 (202/202)	M:35.4 ± 5.1 F:30.9 ± 4.3	QoLICQ	M:200.2 ± 18.44 F:169.54 ± 16.92	CD-RISC	M:65.66 ± 15.45 F:61.64 ± 15.34	CA&MLR	0.13/0.04
Bhamani (26)	2020	Pakistan	–	Hospital	Infertile patients	Purposive	668 (334/334)	M:35.53 ± 6.72 F:30.87 ± 6.12	FertiQoL	M:81.58 ± 12.15 F:70.48 ± 15.69	RS-14	M:77.64 ± 8.56 F:76.19 ± 8.69	MLR	M:−/−8.407 F:−/−8.606
Ha (8)	2020	South Korea	Aug. 2018–Oct. 2018	Hospital Website	Infertile couples	Convenience	300 (150/150)	M:35.81 F:34.03	FertiQoL	M:76.89 ± 12.80 F:71.70 ± 11.41	RS	M:120.36 ± 12.31 F:111.26 ± 16.61	APIM	−/0.201^a^ −/0.713^b^ −/0.219^c^ −/0.351^d^
Li (16)	2019	China	Dec. 2017–Feb. 2018	Hospital	Infertile women	–	498 (0/498)	32.19 ± 3.83	FertiQoL	64.54 ± 16.90	CD-RISC	59.53 ± 16.18	CA&MLR	0.535/0.302
Herrmann (27)	2011	Germany	Mar. 2003–Aug. 2003	Hospital	Infertile couples	–	398 (199/199)	M:35.6 F:33.0	WHOQOL-BREF	–	RS	–	CA	M:0.28/− F:0.33/−
Royani (29)	2019	Iran	Mar. 2015–Jun. 2015	Hospital	Infertile couples	Convenience	404 (202/202)	–	QoLICQ	184 ± 23.36	CD-RISC	63.65 ± 15.51	CA&MLR	0.13/0.04

M, male; F, female; QoL, quality of life; Dec., December; CS, cross-sectional; RIF, repeated implantation failure; FertiQoL, fertility quality of life; CD-RISC, Connor–Davidson resilience scale; MLR, multiple linear regression; CA, correlation analysis; CD-RISC-10, 10-item Connor–Davidson resilience scale; LCA, latent class analysis; PCOS, polycystic ovary syndrome; WHOQOL-BREF, The World Health Organization Quality of Life-BREF; MM, mediation model; APIM, actor–partner interdependence model; RS-14, The 14-item Resilience Scale; RS, the resilience scale; QoLICQ, quality of life questionnaire for infertile couples.

a FertiQoL and resilience of wives.

b FertiQoL and resilience of husbands.

c FertiQoL of husbands and resilience of wives.

d FertiQoL of wives and resilience of husbands.

**Table 2:** Important findings and quality of included studies.

Author/Year/Country	Important findings	Quality scores	Quality ratings
Jiang et al./2022/China (18)	Patients with RIF in ART have low psychological resilience and poor QoL. The family income, the number of hospitals visited, and resilience were independent risk factors of QoL in patients with RIF	4	Moderate
Lian et al./2022/China (10)	FertiQoL is closely related to stigma and resilience in patients with RIF. The higher the level of stigma and the lower the level of resilience, the worse the FertiQoL	5	Moderate
Xie et al./2022/China (31)	The resilience of asthenospermia patients is positively correlated with the FertiQoL, and the resilience and the FertiQoL are in the middle level	6	Moderate
Zhou et al./2022/China (33)	The FertiQoL in infertile men is not optimistic. The more family income and stronger resilience were associated with better FertiQoL, while the worse semen quality, more serious alexithymia, and sexual stress, the worse the FertiQoL	4	Moderate
Mo/2021/China (5)	The level of resilience in infertile women is low, and their resilience has distinct grouping characteristics. Each subgroup has different sociodemographic and disease-related characteristics, such as education level, years of marriage, type of infertility, and fear or helplessness. Moreover, different resilience subgroups have different QoL	8	High
Zhou et al./2023/China (1)	The FertiQoL of infertile patients is low. During treatment, gender, residence, history of accepting ART, and resilience are related to FertiQoL	4	Moderate
Dong et al./2021/China (6)	Psychological resilience and social support have mediating effects on psychological stress and QoL of patients with PCOS	6	Moderate
Liu et al./2020/China (13)	The levels of resilience and QoL of female patients with RIF are lower than those of general infertility. There is a positive correlation between resilience and FertiQoL, and resilience is an influencing factor of FertiQoL	6	Moderate
Zhou et al./2020/China (14)	The resilience of infertility patients is positively correlated with the FertiQoL. The higher the level of resilience of infertility patients, the higher the FertiQoL	6	Moderate
Liu/2019/China (30)	Fertility-related stress, self-efficacy, and resilience are important influencing factors of QoL in female infertility patients. Fertility-related stress can directly predict QoL in female infertility patients. Fertility-related stress is a negative factor for QoL. At the same time, self-efficacy and resilience play a mediating role in the relationship between fertility-related stress and QoL in female infertility patients	9	High
Zhang/2019/China (4)	Resilience significantly affected the FertiQoL of infertile patients	3	Low
Zhou et al./2019/China (34)	The fertiQoL is lower in infertile women. There is a positive correlation between psychological resilience, positive emotion, and FertiQoL in infertile women. History of childbirth, causes of infertility, psychological resilience, and positive emotion are important influencing factors of fertility quality of life in infertile women	6	Moderate
Zhang/2017/China (35)	Resilience and posttraumatic growth could both positively predict QoL. Resilience was the mediator between posttraumatic growth and QoL	8	High
Wang/2022/China (25)	Husbands have higher QoL, resilience, and marital adjustment than wives. Wives’ resilience positively predict wives’ QoL. Wives’ resilience positively predict husbands’ QoL	7	Moderate
Bhamani et al./2022/Pakistan (23)	Among couples, resilience, and QoL were significantly low among wives compared with husbands	8	High
Vatanparast et al./2022/Iran (12)	Low resilience status in infertile couples is better to be considered as a risk factor compromising the QoL	4	Moderate
Bhamani et al./2020/Pakistan (26)	FertiQoL of men and women has a significant association with no formal education, number of friends, income, depression, and resilience	3	Low
Ha et al./2020/South Korea (8)	The resilience of an infertile actor affects both his/her own QoL and his/her partner’s QoL	6	Moderate
Li et al./2019/China (16)	Women with infertility in China had relatively low FertiQoL scores. Resilience influenced the association of infertility-related stress with FertiQoL	8	High
Herrmann et al./2011/Germany (27)	For infertile couples, resilience can be considered an unspecific protective factor against infertility-specific distress and impaired QoL	9	High
Royani et al./2019/Iran (29)	Resilience, gender, and education predict the QoL of infertile couples	3	Low

RIF, repeated implantation failure; ART, assisted reproductive technology; QoL, quality of life; FertiQoL, fertility quality of life; PCOS, polycystic ovary syndrome.

### 3.2 Quality of life

All of the included researches looked into the quality of life of infertile patients. The FertiQoL was utilized in 17 studies (4, 8, 11, 19–28, 30–33), the WHOQOL-BREF in 2 studies (12, 29), and the QoLICQ in 2 studies (6, 10). There were 17 studies that discussed the current situation. In 12 studies, impaired scores were discovered (6, 8, 19, 20, 23–25, 27, 30–33). Three studies, however, found higher QoL scores than before (22, 25, 26). Furthermore, 7 studies found that male patients had higher QoL scores than female patients (6, 8, 10, 11, 20, 30, 31). According to 2 studies, infertile women had a higher score in the social domain and a lower score in the tolerability domain (24, 25). Only one study found that infertile women had higher physical domain ratings (6).

### 3.3 Resilience

All of the included studies looked at the resilience of infertile population. There were 15 studies that utilized the CD-RISC (4, 6, 10, 12, 19–23, 25–27, 31–33), 2 studies that used the CD-RISC-10 (24, 30), 2 studies that used the RS-14 (11, 28), and 2 studies that used the RS (8, 29). There were 10 papers that discussed the current situation. Impaired scores were found in 4 studies (26, 27, 31, 32), and one study indicated a medium level of resilience (26, 27, 31, 32). However, one study reported positive results (29). Furthermore, 4 studies found that male patients were more resilient than female patients (6, 8, 11, 28). In terms of resilience domains, 2 studies reported higher scores in self-improvement and lower scores in optimistic (25, 26).

### 3.4 Association between QoL and resilience

Through correlation analysis and multiple linear regression, 20 studies discovered a favorable relationship between QoL and resilience (4, 6, 10–12, 19–33). One study employed latent class analysis to identify potential resilience categories in infertile women and to examine differences in QoL across subgroups (24). There were 3 potential subgroups in this study: “low resilience group (C1),” “high resilience group (C2),” and “general resilience-low strength group (C3).” In the total score of QoL for each dimension, C2 scored the highest and C1 scored the lowest. Three studies used the mediation model and discovered that resilience mediated psychological stress (12), fertility-related stress (22), post-traumatic growth (25) and QoL, in that order. These studies have indicated that resilience may not directly influence QoL, but it does exert an indirect impact through the medium of social support. The actor-partner interdependence paradigm was the topic of 2 studies. One of them found that an infertile actor’s resilience affects both his/her own and his/her partner’s QoL (8). Another study, however, found that only the resilience of wives can predict the QoL of both couples (30).

### 3.5 Quality assessment

The researches covered in this review ranged in quality from low to high. All 21 studies met at least 3 of the 11 criteria on the AHRQ quality assessment tool and the mean score was 5.86 (SD 1.93). Three studies received a low rating (10, 26, 28), while 6 received a high rating (10, 26, 28). The assessed studies have two common methodological flaws. Firstly, 11 studies failed to justify their sample size and failed to explain the smallest sample size required for appropriate statistical testing (4, 6, 10, 19, 20, 22, 23, 26, 27, 31, 32). Secondly, none of the 10 studies examined the characteristics of nonresponders or tested for non-response bias (4, 6, 10, 19, 20, 22, 23, 26, 27, 31, 32).

## 4 Discussion

Infertility has evolved into a public health issue that requires immediate action. It is inextricably linked to the mental health and quality of life of patients. Previous findings are inconclusive, which may be attributable, at least in part, to variations in the scales and subjects utilized in these studies. Consequently, it is imperative to conduct a comprehensive review of the existing literature on QoL and resilience in infertile patients. This review summarized 21 studies including 7,892 patients, with 2,678 males and 5,214 females. Infertile women always receive more attention than infertile men. Our findings revealed that resilience is not only connected to QoL but also has a significant mediating effect on the association between various psychological markers (such as post-traumatic growth, psychological stress, and so on) and QoL.

The outcomes of this review on levels of QoL and resilience in infertile patients were varied (see Tables 1, 2). QoL was measured using three different tools: FertiQoL, WHOQOL-BREF, and QoLICQ (1998), (7, 16). To test resilience, four different assessment instruments were used: CD-RISC, CD-RISC-10, RS-14, and RS (9, 11, 17, 18). Only 3 studies reported better QoL using the FertiQoL (22, 25, 26), and one study reported better resilience using RS (29). Based on the similarities and differences in domains of each scale, we hypothesize that differences in measurement tools between studies aren’t related to the heterogeneity of findings.

Considerable differences in population among studies may explain the heterogeneity of findings. This finding can be explained by the fact that highly educated patients and patients who live in cities are less likely to be influenced by traditional beliefs due to greater social resources (26). This could also be related to the fact that patients who are further along in the treatment cycle have less doubt and more hope than those who are still in the early stages (22, 25). Cross-cultural differences could potentially explain contradictory conclusions. Asiatic infertile patients showed lower levels of resilience whereas European patients in Germany demonstrated higher levels of resilience (29). These contradictory findings may be explained by the traditional concept of carrying on the family line in Asian. However, the effects of these elements necessitate additional investigation and evidence summarization.

In comparison to infertile women, men have greater QoL and resilience scores (6, 8, 10, 11, 20, 28, 30, 31). This is related to female patients’ higher social and treatment pressures, implying that we should pay more attention to their mental health. Previous research has suggested a correlation between elevated externally oriented thinking in males relative to their partners and their QoL levels. Furthermore, the QoL of both partners appears to be intricately interconnected (35). This finding underscores the significance of implementing interventions aimed at couples as a collective unit. Our review discovered that patients’ treatment tolerability domain and optimistic domain scored low (6, 24–26), indicating that infertility-related treatment has a more profound impact on patients’ lives, and patients’ attitude toward disease treatment is also more pessimistic. As a result, it is critical to pay attention to patients’ psychological status throughout infertility treatment and aim to improve the quality of services provided during treatment, such as the provision of readily available health education, particularly for women.

This review included papers that used correlation analysis, multiple linear regression analysis, and latent class analysis to investigate the relationship between QoL and resilience in infertile couples (4, 6, 10–12, 19–33). This suggests that we should tailor intervention strategies to individual differences in mental resilience in order to improve patients’ quality of life. In two research, the actor-partner interdependence model was applied. One of them discovered that the resilience of an infertile actor affects both his/her own and his/her partner’s QoL (8). However, another study discovered that only the resilience of wives can predict the QoL of both couples (30). Although the results differ, they do present us with a unique perspective on QoL therapies, namely interventions for spouses/interventions for both couples.

Several studies, in contrast to the typical correlation analysis, use the mediation model, which revealed that resilience partially mediated psychological stress (12), fertility-related stress (22), post-traumatic growth (22), and QoL, in that order. One of the studies found that while resilience does not directly affect QoL in patients with polycystic ovary syndrome (PCOS), it does affect QoL indirectly through social support (12). This demonstrates that resilience can mitigate the detrimental impact of stress on QoL while enhancing the good impact of positive stress cognition on QoL. This partially explains the internal mechanism of the positive connection between resilience and QoL and stresses the importance of resilience intervention.

A strength of this review is the adherence to a strict systematic review process and well specified inclusion and exclusion criteria. To find all relevant publications, a comprehensive search approach was used. Another point in favor is the strong inter-rater reliability among reviewers during the screening phase, which reflects transparent selection approach. The current review additionally considered a variety of study approaches and thoroughly synthesizes the literature on QoL and resilience. Finally, this review examined both male and female infertility patients and analyzed the differences between the two, providing future clinical practice with a unique viewpoint to improve the physical and mental health of infertile patients. While this review explored QoL and resilience, it did not look at contributing factors that may have an impact on the balance of outcomes.

This review identified many inherent limitations of related literature. Firstly, the main sampling approach utilized in the included literature is convenience sampling, and there is no guarantee that these patients are a representative sample of all infertile population. Secondly, QoL and resilience were assessed using self-report. As with all self-reports, common methodological variance, social desirability biases, and response distortion due to ego-related defensive tendencies cannot be ignored (36). Not all studies explained any patient exclusions from analysis, and confounding factors were not taken into account. Furthermore, the handling of missing data is ambiguous, and the AHRQ quality assessment revealed poorly reported response rates. Relevant studies are cross-sectional, and the absence of follow-up made investigating cohort effects unfeasible. Finally, this review included only published studies, which will result in an inflated impression of the literature because published studies have more positive results.

## 5 Conclusion

Resilience can significantly predict the QoL of infertile patients. It seems plausible that more resilient couples will be less vulnerable to the stress of infertility. Methodological and population differences across studies may explain the variation in literature results. Future research could focus on broader concepts that include not only mental health but also physical and social well-being. Longitudinal analyses may also be utilised to infer causal relationships between the correlates of mental health, and to explore the changes in the psychological perspective of individuals during the reproductive process. A country’s cultural environment may influence QoL and resilience in infertility. A global consortium of infertile population research could make cross-cultural comparisons of QoL and resilience possible. Future research should concentrate on resilience therapies that improve QoL and alleviate psychological burdens in infertile women.

## Data availability statement

The original contributions presented in the study are included in the article/Supplementary material, further inquiries can be directed to the corresponding authors.

## Author contributions

KL: Conceptualization, Methodology, Writing – original draft. SD: Data curation, Writing – original draft. WQ: Formal analysis, Writing – original draft. DZ: Formal analysis, Writing – original draft. WZ: Visualization, Writing – review & editing. DW: Visualization, Writing – review & editing. CZ: Project administration, Writing – review & editing. YG: Supervision, Writing – review & editing. PT: Supervision, Writing – review & editing.
