# Supplementary material for: Association between quality of life and resilience in infertile patients: a systematic review
Source: Front Public Health. 2024 Feb 27;12:1345899. doi: 10.3389/fpubh.2024.1345899 (PMC10927801; doi:10.3389/fpubh.2024.1345899)
Supplement: Supplementary file 1 [file Table_1.DOCX]

**Table S1 Search formula for each database**

| **Database** | **Search formula** |
| --- | --- |
| **CNKI** | SU=('心理韧性'+'心理弹性'+'复原力'+'心理压弹'+'抗压力'+'抗逆力'+'心理恢复力'+'耐挫力'+'心理资本'+'心理能力'+'脆弱性') AND SU=('生活质量'+'生命质量'+'生存质量') AND SU=('不孕'+'不育'+'不孕不育'+'辅助生殖'+'助孕'+'体外授精'+'胚胎移植'+'冻胚'+'试管婴儿'+'人工授精'+'卵泡质内单精子显微注射'+'卵泡浆内单精子显微注射'+'植入前遗传'+'供精'+'供卵'+'AID'+'AIH'+'IVF'+'ICSI'+'FET'+'PGT'+'PGS'+'PGD'+'ART') |
| **Wanfang data** | 主题:("心理韧性"or"心理弹性"or"复原力"or"心理压弹"or"抗压力"or"抗逆力"or"心理恢复力"or"耐挫力"or"心理资本"or"心理能力"or"脆弱性") and 主题:("生活质量"or"生命质量"or"生存质量") and 主题:("不孕"or"不育"or"不孕不育"or"辅助生殖"or"助孕"or"体外授精"or"胚胎移植"or"冻胚"or"试管婴儿"or"人工授精"or"卵泡质内单精子显微注射"or"卵泡浆内单精子显微注射"or"植入前遗传"or"供精"or"供卵"or"AID"or"AIH"or"IVF"or"ICSI"or"FET"or"PGT"or"PGS"or"PGD"or"ART") |
| **VIP database** | U=(心理韧性 OR 心理弹性 OR 复原力 OR 心理压弹 OR 抗压力 OR 抗逆力 OR 心理恢复力 OR 耐挫力 OR 心理资本 OR 心理能力 OR 脆弱性) AND U=(生活质量 OR 生命质量 OR 生存质量) AND U=(不孕 OR 不育 OR 不孕不育 OR 辅助生殖 OR 助孕 OR 体外授精 OR 胚胎移植 OR 冻胚 OR 试管婴儿 OR 人工授精 OR 卵泡质内单精子显微注射 OR 卵泡浆内单精子显微注射 OR 植入前遗传 OR 供精 OR 供卵 OR AID OR AIH OR IVF OR ICSI OR FET OR PGT OR PGS OR PGD OR ART) |
| **PubMed** | ((resilience[Title/Abstract]) OR (resilient[Title/Abstract]) OR (resiliency[Title/Abstract]) OR (psychological adaptation[Title/Abstract]) OR (elasticity[Title/Abstract]) OR (recovery[Title/Abstract]) OR (mental toughness[Title/Abstract])) AND ((quality of life[Title/Abstract]) OR (QOL[Title/Abstract])) AND ((infertility[Title/Abstract]) OR (sterility[Title/Abstract]) OR (subfertility[Title/Abstract]) OR (assisted reproductive technology[Title/Abstract]) OR (ART[Title/Abstract]) OR (in vitro fertilization[Title/Abstract]) OR (IVF[Title/Abstract]) OR (intracytoplasmic sperm injection[Title/Abstract]) OR (ICSI[Title/Abstract]) OR (preimplantation genetic diagnosis[Title/Abstract]) OR (preimplantation genetic testing[Title/Abstract]) OR (preimplantation genetic screening[Title/Abstract]) OR (insemination[Title/Abstract]) OR (PGT[Title/Abstract]) OR (PGD[Title/Abstract]) OR (PGS[Title/Abstract]) OR (AID[Title/Abstract]) OR (AIH[Title/Abstract]) OR (FET[Title/Abstract]) OR (donor sperm[Title/Abstract]) OR (donor semen[Title/Abstract]) OR (donor egg[Title/Abstract]) OR (donor oocyte[Title/Abstract]) OR (embryo[Title/Abstract]) OR (blastocyst[Title/Abstract])) |
| **Web of Science** | AB=(resilience OR resilient OR resiliency OR psychological adaptation OR elasticity OR recovery OR mental toughness) AND AB=(quality of life OR QOL) AND AB=(infertility OR sterility OR subfertility OR assisted reproductive technology OR ART OR in vitro fertilization OR IVF OR intracytoplasmic sperm injection OR ICSI OR preimplantation genetic diagnosis OR preimplantation genetic testing OR preimplantation genetic screening OR insemination OR PGT OR PGD OR PGS OR AID OR AIH OR FET OR donor sperm OR donor semen OR donor egg OR donor oocyte OR embryo OR blastocyst) |
| **Embase** | (resilience:ab,ti OR resilient:ab,ti OR resiliency:ab,ti OR 'psychological adaptation':ab,ti OR elasticity:ab,ti OR recovery:ab,ti OR 'mental toughness':ab,ti) AND ('quality of life':ab,ti OR qol:ab,ti) AND (infertility:ab,ti OR sterility:ab,ti OR subfertility:ab,ti OR 'assisted reproductive technology':ab,ti OR art:ab,ti OR 'in vitro fertilization':ab,ti OR ivf:ab,ti OR 'intracytoplasmic sperm injection':ab,ti OR icsi:ab,ti OR 'preimplantation genetic diagnosis':ab,ti OR 'preimplantation genetic testing':ab,ti OR 'preimplantation genetic screening':ab,ti OR insemination:ab,ti OR pgt:ab,ti OR pgd:ab,ti OR pgs:ab,ti OR aid:ab,ti OR aih:ab,ti OR fet:ab,ti OR 'donor sperm':ab,ti OR 'donor semen':ab,ti OR 'donor egg':ab,ti OR 'donor oocyte':ab,ti OR embryo:ab,ti OR blastocyst:ab,ti) |
